# A traffic light control method based on multi-agent deep reinforcement learning algorithm

**DOI:** 10.1038/s41598-023-36606-2

**Published:** 2023-06-09

**Authors:** Dongjiang Liu, Leixiao Li

**Affiliations:** College of Data Science and Application, Inner Mongolia University of Technology, Inner Mongolia Autonomous Region Engineering and Technology Research Center of Big Data Based Software Service, Huhhot, 10080 Inner Monglia China

**Keywords:** Computer science, Information technology, Scientific data

## Abstract

Intelligent traffic light control (ITLC) algorithms are very efficient for relieving traffic congestion. Recently, many decentralized multi-agent traffic light control algorithms are proposed. These researches mainly focus on improving reinforcement learning method and coordination method. But, as all the agents need to communicate while coordinating with each other, the communication details should be improved as well. To guarantee communication effectiveness, two aspect should be considered. Firstly, a traffic condition description method need to be designed. By using this method, traffic condition can be described simply and clearly. Secondly, synchronization should be considered. As different intersections have different cycle lengths and message sending event happens at the end of each traffic signal cycle, every agent will receive messages of other agents at different time. So it is hard for an agent to decide which message is the latest one and the most valuable. Apart from communication details, reinforcement learning algorithm used for traffic signal timing should also be improved. In the traditional reinforcement learning based ITLC algorithms, either queue length of congested cars or waiting time of these cars is considered while calculating reward value. But, both of them are very important. So a new reward calculation method is needed. To solve all these problems, in this paper, a new ITLC algorithm is proposed. To improve communication efficiency, this algorithm adopts a new message sending and processing method. Besides, to measure traffic congestion in a more reasonable way, a new reward calculation method is proposed and used. This method takes both waiting time and queue length into consideration.

## Introduction

Intelligent Traffic Light Control (ITLC) is a good method for relieving traffic congestion. An ITLC algorithm should detect traffic condition of intersection and adjust cycle length of traffic light automatically. As reinforcement learning algorithm^1^ works very well in automatic control^2,3^, many reinforcement learning based ITLC algorithms are proposed recently. These algorithms can be classified into two categories, including tabular methods^4–6^ and approximation methods^7–9^. In approximation methods, *Q*-value of every state-action pair should be calculated by a specific model. Given a state, if an action gets the highest *Q*-value, it will be selected by the agent and performed in outer environment. Generally, a traffic light controller is treated as an agent. They are responsible for collecting states from outer environment, calculating reward value and selecting action. Nowadays, many different models are proposed to fulfil *Q*-value calculation task, including feed-forward neural networks based model^7^, linear model^10^, probabilistic model^11^, convolutional neural networks based model^8^, RAIM model^12^, graph convolutional networks based model^9^, FRAP model^13,14^, MetaLight model^15^ etc. The reinforcement learning algorithms that use deep learning models^16^ to calculate *Q*-value are called deep reinforcement learning algorithms. As deep reinforcement learning algorithms perform very well, they are applied into many fields, including network abnormal traffic detection^17,18^, communications and networking^19^ etc.

Based on the above description, the traffic signal control algorithms can be classified into two types, which are single-agent based algorithms^20,21^ and multi-agent based algorithms^22–24^. In multi-agent based algorithms, all the traffic light controllers of a traffic grid should coordinate to cope with traffic congestion. Multi-agent based algorithms can be classified into two types as well, including centralized algorithms^25–27^ and decentralized algorithms^28–30^. In centralized algorithms, an executor is leveraged to learn the joint action for all the agents. As all the agents are controlled by an center executor, the scalability of centralized algorithm is not as good as expected. Decentralized algorithms is the best option to solve this problem. In decentralized algorithms, every traffic signal controller will be treated as an independent agent and it selects an action by itself. Nowadays several decentralized algorithms are proposed. CGB-MATSC algorithm^28^ is an cluster based traffic signal control algorithm. In this algorithm, all the agents will be clustered into different clusters and each cluster is controlled by an executor. NAQL algorithm^29^ is a decentralized algorithm which employs fuzzy logic to optimize the model. MA2C algorithm^30^ is a multi-agent based traffic signal control algorithm which is based on actor-critic method. Co-DQL algorithm^31^ is proposed based on double *Q*-learning method. Double estimators are used in this algorithm. MPLight algorithm^32^ is proposed to control a thousand traffic lights. It is based on reinforcement learning algorithm and transportation theories. EMVLight algorithm^33^ can perform dynamic routing and traffic light control simultaneously. MARDDPG algorithm^34^ is proposed based on deep deterministic policy gradient algorithm. ATSC algorithm^35^ is a network-level decentralized adaptive signal control algorithm and a deep reinforcement learning is used in this algorithm. From above description, we can find that all these algorithms mainly concentrate on improving model effect and coordination method. But communication details are overlooked. As, when agents try to coordinate with each other, communication will happens among them, a communication method should be proposed to ensure efficiency of coordination. Firstly, an effective traffic condition description method is needed. By using this method, traffic condition of an intersection can be described by a message simply and clearly. This message will be sent to other agents. Secondly, synchronization of the decentralized algorithms should be further improved. Every traffic light controllers need to send message to other at the end of each cycle. But, as cycle length of different traffic light is different, it is not easy for an agent to decide which message is the latest one and the most valuable. To solve these problems, a new message sending and processing method is proposed. In this method, traffic condition can be described by a message simply and clearly. And, at the same time, a data structure is used by the proposed method to record the latest and the most valuable messages for further processing. In addition to the communication details, reinforcement learning algorithm used for traffic light control should also be improved. In traditional algorithms, either queue length or waiting time of congested vehicles is considered while calculating reward value. But, both of these two factors are important for judging traffic congestion. So a new reward value calculation method that take these two factors into consideration is proposed in this paper. Accordingly, the contribution of this paper is as below:

- A decentralized multi-agent reinforcement learning algorithm based intelligent traffic light control method is proposed.
- A new message sending and processing method is proposed. In this method, traffic condition can be described by a message simply and clearly. Besides, a data structure is used by this method to record the latest and most valuable message for further processing.
- A new reward calculation method is proposed. This method takes both queue length and total waiting time into consideration.

The remainder of this paper is organized as follows. The proposed algorithm is described in the second section. The experimental results are presented in the third section. And the last section is the conclusion of this paper.

## Traffic light control algorithm based on multi-agent deep reinforcement learning

### Architecture of the proposed traffic light control method

In this section, the proposed multi-agent based ITLC algorithm will be presented. Two factors are considered by an agent who executes the proposed algorithm. One is the traffic condition of corresponding intersection. The other is traffic condition of neighbor intersections. As the vehicles that appear at the neighbor intersections may arrive at current intersection in the next moment, traffic condition of neighbor intersections should be considered as well. So two steps are executed by the proposed algorithm. Firstly, the agent will collect the traffic condition of corresponding intersection as state. And, then, select a new action $a'$ by using deep reinforcement learning algorithm. Secondly, traffic condition of neighbor intersections will be collected. And cycle length assigned by action $a'$ will be further updated based on the newly collected traffic condition. Then the final cycle length $c^{'}_{f}$ is obtained. It will be executed in the next cycle. The procedure described above will keep repeating. The framework of the proposed algorithm is presented in Algorithm 1.
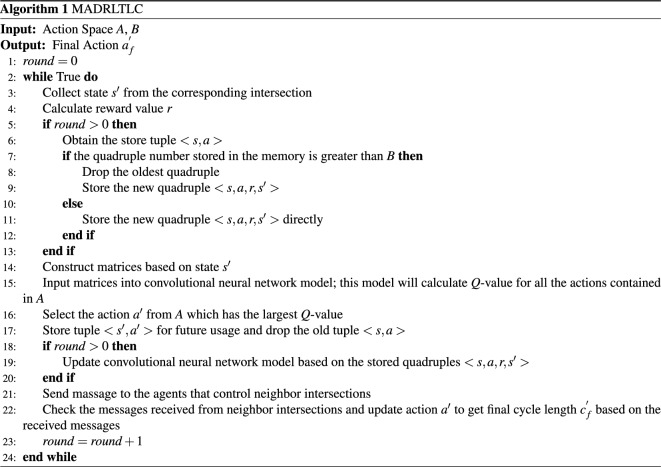


The process described in algorithm 1 is executed by every agent. As traffic signal timing task is executed periodically, the loop condition is set to *True*. Variable *round* is used to represent the cycle number of traffic light. Psuedo-code from line 3 to line 13 describes new quadruple construction procedure. During the procedure, a new state $s'$ is collected and reward value *r* is calculated. The content that introduces state is presented in section States and the content that introduces reward calculation method is presented in section “Reward”. In the new quadruple, *s* is the state collected at the end of the last cycle. *a* is the action selected at the end of last cycle as well. The content that introduces actions is presented in section “Action space”. Psuedo-code from line 14 to line 16 describes the new action selection procedure. During the procedure, a new action $a'$ is selected by using CNN model. Structure of CNN model is presented in section “*Q*-value calculation model”. In line 17 of psuedo-code, tuple $\langle s',a' \rangle$ is stored for future quadruple construction. Psuedo-code from line 18 to line 20 describes parameter training procedure. During the procedure, variable *round* is checked. If its value is greater than 0, it means that at least one quadruple is constructed. So the parameter training process can be executed. Detailed model parameter training procedure is introduced in section “*Q*-value calculation model” as well. In line 21 of psuedo-code, an agent sends messages to its neighbors, which describe traffic condition of corresponding intersection. In line 22 of psuedo-code, this agent checks the messages sent by its neighbors. Based on these messages, final cycle length $c^{'}_{f}$ can be obtained. The detailed communication procedure and cycle length updating procedure is introduced in section “Information exchanging and processing method”. As neighbor agents try to send message at any time, a specific thread should be performed to receive and store messages.

### States

In reinforcement learning algorithm, an agent should collect state from outer environment. While we try to collect state from an intersection, a big square region centered by traffic light will be observed. The distance between boundary and center of this square area is 150 meters long. This big square region can be further divided into smaller squares. Generally, the length of a vehicle is approximately 5 meters. The distance between two vehicles is approximately one meter. So the edge length of each small square should be set to 6 meters. At this time, position matrix and speed matrix can be built based on this big square region. If a small square is occupied by a vehicle, the corresponding element of position matrix should be 1. Otherwise, the corresponding element should be 0. Similarly, if a small square is occupied by a vehicle and the speed of this vehicle is not 0, the corresponding element of speed matrix will be the speed value of this vehicle. If there is no vehicle in the small square or the vehicle speed is 0, the corresponding element of speed matrix will be 0. Obviously, the shape of these two matrices is $50\times 50$.

**Figure 1:**
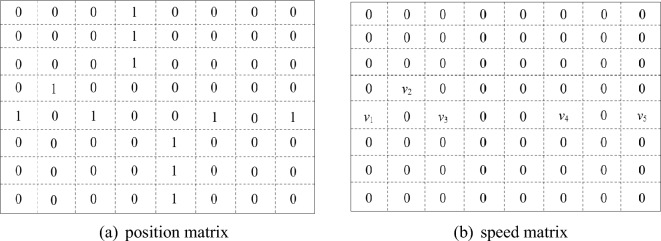
State collected from intersection.

An example of position matrix and speed matrix is presented in Fig. 1. Particularly, position matrix is presented in Fig. 1a and speed matrix is presented in Fig. 1b. We can find that, in Fig. 1a, the element value in row 5, column 1 is 1. Thus corresponding small square contains a vehicle. And, in Fig. 1b, the element value in row 5, column 1 is $v_0$, which is greater than 0. It means that speed of the vehicle contained in this small square is greater than 0. So we can conclude that this vehicle keeps running. Besides, in Fig. 1a, element value of row 1,column 4 is 1. It means that a vehicle is contained in this small square. But, in Fig. 1b, element value of row 1,column 4 is 0. It means that speed of this vehicle is 0. As these two matrices can reflect traffic condition of an intersection, they will be treated as state and input into a *Q*-value calculation model.

### Action space

**Figure 2:**
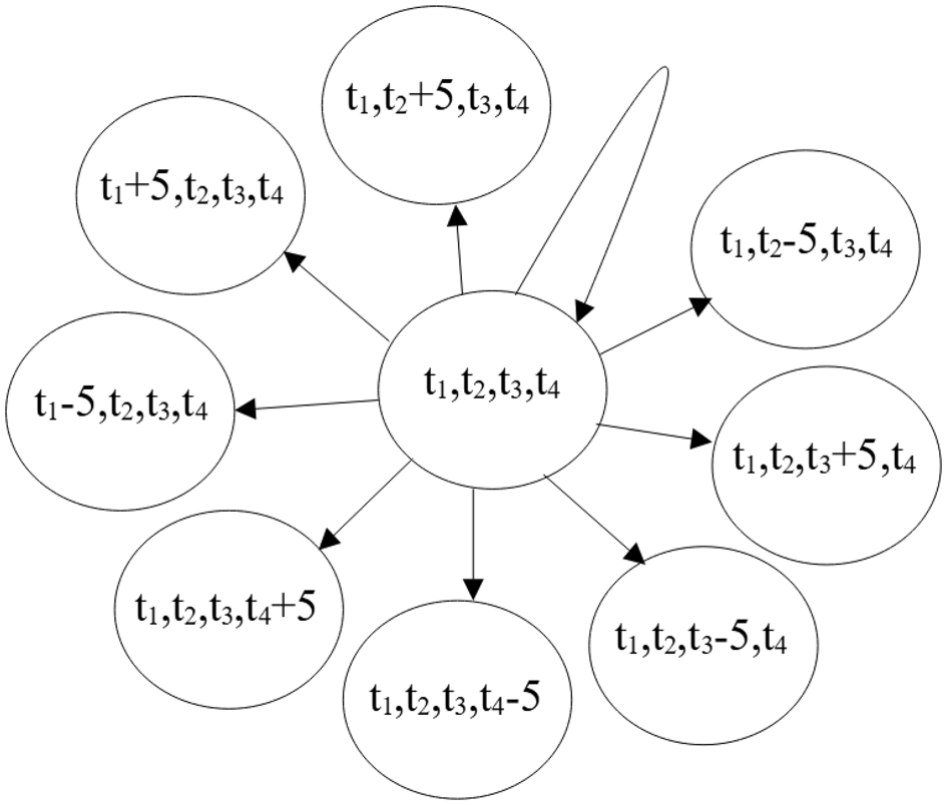
Action space used in the proposed algorithm.

In reinforcement learning algorithm, the task of model is to calculate a *Q*-value for each action. Then action with the highest *Q*-value will be selected and executed in the outer environment. An action space contains all the actions and their transition relationship. The action space used by the proposed ITLC algorithm is presented in Fig. 2.

In the proposed algorithm, four phases are considered. Phase 1 is green light that controls northbound through and southbound through vehicles; Phase 2 is green light that controls southbound left-turn and northbound left-turn vehicles; Phase 3 is green light that controls eastbound through and westbound through vehicles; Phase 4 is green light that controls westbound left-turn and eastbound left-turn vehicles. In all the actions, four values are used, which are $t_1$, $t_2$, $t_3$ and $t_4$. $t_1$ represents the duration of phase 1; $t_2$ represents the duration of phase 2; $t_3$ represents the duration of phase 3; $t_4$ represents the duration of phase 4. In Fig. 2, we can find that updating value of $t_1$, $t_2$, $t_3$ or $t_4$ is the main task of actions. Every action tries to add 5 s to one of these four values or reduced 5 s from one of these four values. And, particularly, an agent can also select an action that doesn’t change the value of $t_1$, $t_2$, $t_3$ and $t_4$. It is noteworthy that the maximum value of $t_1$, $t_2$, $t_3$ and $t_4$ is 90 s. Suppose that the value of $t_4$ is 90. If the action that adds another 5 s to $t_4$ is selected, it won’t be executed. And the minimum value of $t_1$, $t_2$, $t_3$ and $t_4$ is 5. Similarly, if the value of $t_4$ is 5 and the action that reduces another 5 s from $t_4$ is selected, it also won’t be executed.

### Reward

Reward can be used to measure the result of an action. It is calculated based on the variation of outer environment. As reinforcement learning based ITLC algorithms are used to relieve traffic congestion, the reward value must reflect the variation of traffic congestion condition. Two factors are important for evaluating traffic congestion, which are queue length of the congested vehicles and total waiting time of all these vehicles. But traditional ITLC algorithms just take one of them into consideration. If only waiting time is considered, while plenty of vehicles arrive at an intersection and form a long waiting queue in a short time, even though the cumulative waiting time of all these newly arrived vehicles is very short, the queue length is very long. If only queue length is considered, when the queue length of congested vehicles is not very long, the corresponding lane will be justified as non-congestion. Then the phase length will be reduced. In this situation, it is also hard for these vehicles to pass the intersection. So a new reward calculation method should be proposed, which takes both queue length and waiting time into consideration.

Accordingly, the reward calculation method used by the proposed algorithm is as below:

1 $$\begin{aligned} r_t = V_t-V_{t+1} \end{aligned}$$

2 $$\begin{aligned} V_t = l_{NS,SN}*W_{NS,SN}+l_{NE,SW}*W_{NE,SW}+l_{EW,WE}*W_{EW,WE}+l_{ES,WN}*W_{ES,WN} \end{aligned}$$

3 $$\begin{aligned} l_{NS,SN}=&\,max\{l_{NS},l_{SN}\}\nonumber \\ l_{NE,SW}= &\,max\{l_{NE},l_{SW}\}\nonumber \\ l_{EW,WE}= &\,max\{l_{EW},l_{WE}\}\nonumber \\ l_{ES,WN}= & \,max\{l_{ES},l_{WN}\} \end{aligned}$$

4 $$\begin{aligned} W= & {} \sum _{n=1}^{N_{t}} w_{n} \end{aligned}$$

$r_t$ represents reward value. $V_t$ is the value calculated at the end of the $t-th$ cycle of traffic signal. $V_{t+1}$ is the value calculated at the end of the $(t+1)-th$ cycle. The calculation method of value $V_t$ is presented in Eq. (2). In Eq. (2), four queue length are used, including $l_{NS,SN}$, $l_{NE,SW}$, $l_{EW,WE}$ and $l_{ES,WN}$. Take $l_{NS,SN}$ for example. The calculation method of value $l_{NS,SN}$ is presented in the first Eq. of (3). In this equation, $l_{NS}$ represents the queue length of southbound through vehicles. $l_{SN}$ represents the queue length of northbound through vehicles. To ensure the vehicles of these two queues get enough time to pass the intersection, $l_{NS,SN}$ should be set to the maximum value of $l_{NS}$ and $l_{SN}$. From Eq. (3), we can find that $l_{NE,SW}$, $l_{EW,WE}$ and $l_{ES,WN}$ can be calculated in the same way. $l_{NE}$ represents the queue length of southbound left-turn vehicles. $l_{SW}$ represents the queue length of northbound left-turn vehicles. $l_{EW}$ represents the queue length of westbound through vehicles. $l_{WE}$ represents the queue length of eastbound through vehicles. $l_{ES}$ represents the queue length of westbound left-turn vehicles. $l_{WN}$ represents the queue length of eastbound left-turn vehicles. Moreover, in Eq. (2), $W_{NS,SN}$, $W_{NE,SW}$, $W_{EW,WE}$ and $W_{ES,WN}$ are contained. They represents the total waiting time of vehicles of specific direction. The calculation method of these four values is presented in Eq. (4). While $W_{NS,SN}$ is calculated, $N_t$ represents the number of southbound through and northbound through vehicles. Similarly, while $W_{NE,SW}$ is calculated, $N_t$ represents the number of southbound left-turn and northbound left-turn vehicles. While $W_{EW,WE}$ is calculated, $N_t$ represents the number of westbound through and eastbound through vehicles. While $W_{ES,WN}$ is calculated, $N_t$ represents the number of eastbound left-turn and westbound left-turn vehicles. No matter which value is calculated by Eq. (4) , $w_n$ represents the waiting time of $n-th$ vehicle of specific direction.

### *Q*-value calculation model

In model based reinforcement learning algorithms, the model is used to calculate *Q*-value for each action. As the state collected from an intersection is represented by two matrices, convolutional neural network is used by the proposed algorithm to calculate *Q*-value for action.

**Figure 3:**
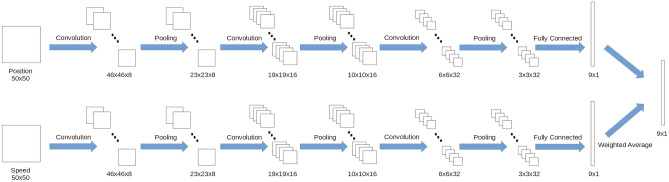
Convolutional neural network model used in the proposed algorithm.

Convolutional neural network (CNN) model used by the proposed algorithm is presented in Fig. 3. We can find that position matrix and speed matrix are input into same convolutional neural network model separately. Then two different vectors are obtained. Each one contains 9 values. Suppose that these two vectors are $vec_p$ and $vec_s$. Based on these two vectors, a new vector is calculated. It also contains 9 values. This new vector is called $vec_n$. As 9 actions are contained in action space, each element of $vec_n$ is corresponding to an action. Method used to calculate element value of $vec_n$ is presented in Eq. (5).

5 $$\begin{aligned} n_i=0.6*p_i+0.4*s_i \end{aligned}$$

$n_i$ is the $i-th$ element of $vec_n$. $p_i$ is $i-th$ element of $vec_p$. And $s_i$ is $i-th$ element of $vec_s$. In Eq. (5), we can find that the weight value of $p_i$ is higher. It is because that the position of vehicles is more important than the speed of vehicles for judging traffic congestion. If queue of congested vehicles is very long, even though the congested vehicles are running, it is not easy for them to pass the intersection in a short period. But, obviously, if congested vehicles keep running, the traffic congestion can be relieved. So the speed of congested vehicles should be also considered. Thus, both $p_i$ and $s_i$ are used while calculating $n_i$. At the same time, the weight value of $p_i$ is greater than $s_i$.

In the CNN model presented in Fig. 3, three convolutional layers are contained. The first convolutional layer contains 8 filters. The second convolutional layer contains 16 filters. The third convolutional layer contains 32 filters. Each filter’s size is $5\times 5$ and it moves $1\times 1$ stride every time through the input data. Moreover, three pooling layers are contained in the model. Max pooling method is used. And size of filters contained in pooling layers is $2\times 2$. Output size of the first convolutional layer is $46\times 46\times 8$. Output size of the first pooling layer is $23\times 23\times 8$. Output size of the second convolutional layer is $19\times 19\times 16$. Output size of the second pooling layer is $10\times 10\times 16$. Output size of the third convolutional layer is $6\times 6\times 32$. Output size of the third pooling layer is $3\times 3\times 32$. The output of the third pooling layer will be transformed into a tensor. The shape of this tensor is $288\times 1$. After that, this tensor is input into a fully connected layer. The output of fully connected layer is a vector, which contains 9 values. Fully connected layer contains two different layers. In the first layer, 100 neurons are contained. And, in the second layer, 9 neurons are contained. ReLU function is the activation function used in the model described above.

Initially, parameters of the CNN model are randomly assigned. Then they will be trained continuously. In the proposed algorithm, parameter training procedure is executed at the end of each traffic light cycle. Generally, the main target of reinforcement learning is to select a series of actions that can make outer environment reach the optimal state. This requirement can be depicted by Eq. (6).

6 $$\begin{aligned} Q(s,a)=R(s,a)+\gamma Q\left( s',\mathop {argmax}\limits _{a'}\left( s',a'\right) \right) \end{aligned}$$

In Eq. (6), *s* represents the last state. *a* represents the last selected action. *Q*(*s*, *a*) is the *Q*-value obtained by performing action *a* given state *s*. *R*(*s*, *a*) represents the reward. $s'$ represents the newly collected state after performing action *a*. $a'$ is the newly selected action. After performing action $a'$ based on state $s'$, the maximum *Q*-value can be obtained. This maximum *Q*-value is $Q(s',\mathop {argmax}\limits _{a'}(s',a'))$. $\gamma$ is discount factor. Based on the requirement presented in Eq. (6), the loss function used by the proposed algorithm to train CNN model is presented in Eq. (7).

7 $$\begin{aligned} J=\sum _{s} \frac{1}{B}\left[ R(s,a)+\gamma Q\left( s',\mathop {argmax}\limits _{a'}\left( s',a'\right) \right) -Q(s,a)\right] ^2 \end{aligned}$$

*B* represents the number of quadruples constructed and stored before. In Eq. (7), meaning of *R*(*s*, *a*), $Q(s',\mathop {argmax}\limits _{a'}(s',a'))$, *Q*(*s*, *a*) and $\gamma$ is same with Eq. (6). From Eq. (6), we know that $R(s,a)+\gamma Q(s',\mathop {argmax}\limits _{a'}(s',a'))$ and *Q*(*s*, *a*) should be as close as possible. So the loss function need to be minimized. This loss function can be optimized by using ADAptive Moment estimation (Adam) method.

**Figure 4:**
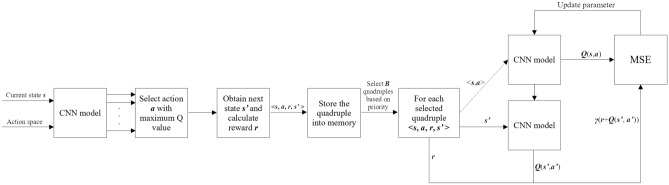
Loss function value calculation procedure.

During the optimization procedure, the value of loss function should be calculated. The calculation procedure is presented in Fig. 4. During the procedure, the stored quadruples are used. These quadruples are in the form of $\langle s,a,r,s' \rangle$. Quadruple collection procedure is presented in the first three steps of Fig. 4. Loss function value calculating task also contains three steps. Firstly, calculate *Q*(*s*, *a*) based on $\langle s,a \rangle$. Secondly, calculate the highest *Q*-value $Q(s',a')$ based on state $s'$ and CNN model. The first step and the second step should repeat *B* times and *B* different quadruples will be used. Thirdly, calculate the value of loss function presented in Eq. (7) by using all the *Q*(*s*, *a*), *r* and $Q(s',a')$ calculated before.

### Information exchanging and processing method

Generally, traffic condition of an intersection can impact its neighbors. It is because vehicles that appear at an intersection will arrive at its adjacent intersections at the next moment. So traffic congestion of an intersection can spread to its neighbors in the nearly future. In this situation, to relieve future traffic congestion, every agent should communicate with its neighbors by sending messages. The messages contain traffic condition information of corresponding intersection. The proposed communication method is described in this section.

As every agent should select an action based on the its local traffic condition, the selected action can be used to represent the traffic condition. In this situation, an agent can try to observe the traffic condition of its neighbors based on the action selected by its neighbors. Thus the message sent to neighbors must contains two factors. One is the agent location information. The other is action type. So the message should be in the form of $\langle direction,action\_type \rangle$. *direction* represents which direction the message is sent to. So it will be used to present the location of message sender. The value of *direction* can be *n*, *s*, *e* and *w*, which mean north, south, east and west. The value of $action\_type$ can be 0, 1 or $-1.0$ means that the specific phase length of message sender is keep unchanging. 1 means that the 5 s is added to the related phase length. $-1$ means that 5 s is reduced from the related phase length. According to this, we can find that different selected actions corresponds to different messages. Corresponding relationship between actions and messages is presented in Table 1. If an action presented in the first column is selected, messages presented in the second column will be sent to the corresponding neighbors. For example, while action $\langle t_1+5,t_2,t_3,t_4 \rangle$ is selected, $\langle n,1 \rangle$ will be sent to the north neighbor and $\langle s,1 \rangle$ will be sent to the south neighbor. $\langle e,0 \rangle$ will be sent to the east neighbor. $\langle w,0 \rangle$ will be sent to the west neighbor.

**Table 1:** Corresponding relationship between actions and messages.

$\langle t_1+5,t_2,t_3,t_4 \rangle$	$\langle n,1 \rangle , \langle s,1 \rangle , \langle e,0 \rangle , \langle w,0 \rangle$
$\langle t_1-5,t_2,t_3,t_4 \rangle$	$\langle n,-1 \rangle , \langle s,-1 \rangle , \langle e,0 \rangle , \langle w,0 \rangle$
$\langle t_1,t_2+5,t_3,t_4 \rangle$	$\langle n,0 \rangle , \langle s,0 \rangle , \langle e,1 \rangle , \langle w,1 \rangle$
$\langle t_1,t_2-5,t_3,t_4 \rangle$	$\langle n,0 \rangle , \langle s,0 \rangle , \langle e,-1 \rangle , \langle w,-1 \rangle$
$\langle t_1,t_2,t_3+5,t_4 \rangle$	$\langle n,0 \rangle , \langle s,0 \rangle , \langle e,1 \rangle , \langle w,1 \rangle$
$\langle t_1,t_2,t_3-5,t_4 \rangle$	$\langle n,0 \rangle , \langle s,0 \rangle , \langle e,-1 \rangle , \langle w,-1 \rangle$
$\langle t_1,t_2,t_3,t_4+5 \rangle$	$\langle n,1 \rangle , \langle s,1 \rangle , \langle e,0 \rangle , \langle w,0 \rangle$
$\langle t_1,t_2,t_3,t_4-5 \rangle$	$\langle n,-1 \rangle , \langle s,-1 \rangle , \langle e,0 \rangle , \langle w,0 \rangle$
$\langle t_1,t_2,t_3,t_4 \rangle$	$\langle n,0 \rangle , \langle s,0 \rangle , \langle e,0 \rangle , \langle w,0 \rangle$

As different agents will send messages at different time, synchronization of the messages should be considered. It is fulfilled by using a data structure. While a message is received by an agent, this message will be stored. The data structure used to store the received message is in the form of $(\langle n,0 \rangle ,\langle s,0 \rangle ,\langle e,0 \rangle ,\langle w,0 \rangle )$. It contains four tuples. Initially, second elements of all the tuples are 0. They will be updated based on the received messages. For example, if $\langle n,1 \rangle$ is received, the data structure will becomes $(\langle n,1 \rangle ,\langle s,0 \rangle ,\langle e,0 \rangle ,\langle w,0 \rangle )$. While a new message is received, the data structure need to be updated immediately. By doing so, the real-time messages can be stored in this data structure in order to replace the old messages. While performing traffic signal timing, after a new action $a'$ is selected, the data structure should be checked. Suppose that the data structure is changed to $(\langle n,value_1 \rangle , \langle s,value_2 \rangle , \langle e,value_3 \rangle , \langle w,value_4 \rangle )$. If either $value_1$ or $value_2$ is 1, $t_1$ and $t_2$ of action $a'$ will be added another 3 s. If the value of $value_1$ and $value_2$ are (0, 0), $(0,-1)$ or $(-1,0)$, $t_1$ and $t_2$ will keep unchanging. If both $value_1$ and $value_2$ are $-1$, 3 s will be reduced from $t_1$ and $t_2$. If either $value_3$ or $value_4$ is 1, $t_3$ and $t_4$ of action $a'$ will be added another 3 s. If the value of $value_3$ and $value_4$ are (0, 0), $(0,-1)$ or $(-1,0)$, $t_3$ and $t_4$ will keep unchanging. If both $value_3$ and $value_4$ are -1, 3 minutes will be reduced from $t_3$ and $t_4$. While updating procedure is finished, data structure will be reset to $(\langle n,0 \rangle ,\langle s,0 \rangle ,\langle e,0 \rangle ,\langle w,0 \rangle )$.

### Algorithm complexity analysis

In algorithm 1, we can find that all the steps are contained in a while loop. This loop is an infinite iteration. The running time of this loop depends on the number of traffic light cycle. If *n* cycles have been executed by a traffic light, the while loop of algorithm 1 is performed *n* times. Among all the steps contained in while loop, the most time consuming step is model training procedure. This task is fulfilled by using Adam algorithm. As the complexity of Adam algorithm is $O(\log {d\sqrt{T}})$, if *n* cycles have been performed by a traffic light, the complexity of the proposed algorithm is $O(n*\log {d\sqrt{T}})$.

## Evaluation

### Datasets

Two simulation experiments are executed. Every simulation experiment contains a trajectory dataset and a road network. In the first experiment, synthetic trajectory data is used. In the second experiment, real-world trajectory data is used. The features of these two datasets and corresponding road network will be introduced below.

**Synthetic trajectory dataset:** This dataset is created artificially. It contains the trajectory of 5372 vehicles. The arrival rate of the generated vehicles obeys Gaussian distribution. Running speed of all the vehicles is limited below 55km/h. And, among all these vehicles, 20% of them choose to turn right; 60% of them choose to go straight; and rest vehicles choose to turn left. The constructed road network contains 9 intersections. It is a $3 \times 3$ grid. So 3 intersections are contained in each column and each row.

**Real-world trajectory dataset:** This dataset contains the trajectory of 2983 vehicles. And it is collected from Dongfeng subdistrict, Jinan, Shandong province, China. All these vehicles enter into the road network within 1 hour. The constructed road network contains 12 intersections. It is a $3 \times 4$ grid. So there are 4 intersections in each row. And each column contains 3 intersections.

In synthetic trajectory dataset, the traffic grid is a $3 \times 3$ network and there are 5372 vehicles running in this traffic grid. Obviously, the vehicle number is very large. So the efficiency of the proposed ITLC algorithm in relieving heavy traffic congestion can be tested. At the same time, we can find that the synthetic data is different from real-world data. Firstly, there is difference between synthetic trajectories and real-world trajectories. Secondly, the vehicle number of real-world dataset is less than synthetic dataset significantly. Thirdly, the traffic grid of real-world dataset is larger. Thus, to test efficiency of the proposed algorithm in real-world environment, a real-world trajectory dataset is used in the experiment.

### Evaluation metrics

To evaluate the ITLC algorithms, two metric values are used in this experiment, which are average waiting time and average reward value. The reason is described below:

**Average waiting time:** Obviously, one goal of relieving traffic congestion is to reduce the total waiting time of all the congested vehicles. So the more the total waiting time is reduced, the better the ITLC algorithm is. As average waiting time is proportional with total waiting time, average waiting time of congested vehicles will be used as a metric.

**Reward:** To take both waiting time and queue length into consideration, reward value is calculated by using the method presented in Reward section. Average reward value of all the agents is used as a metric. According to the proposed reward calculation method, the higher the average reward value is, the better the ITLC algorithm is.

### Compared algorithms

As the proposed algorithm tries to promote coordination effect of decentralized ITLC algorithm, two other ITLC algorithms that adopt different coordination method should be compared with the proposed algorithm. Among these two algorithms, one is single-agent based algorithm which is called SABA; The other is multi-agent based decentralized algorithm which is called MARDDPG^34^. These two algorithms are described below.

**SABA:** In this algorithm, deep Q-network (DQN) algorithm is used to control every traffic light. The deep neural network used in the DQN is convolutional neural network. State, action spaces and reward calculation method used by SABA are same with the proposed ITLC algorithm. But the communication procedure among agents is eliminated by this algorithm. So all the agents work independently.

**MARDDPG:** MARDDPG is a multi-agent based decentralized algorithm. This algorithm is proposed based on deep deterministic policy gradient algorithm. It tries to train the deep neural network model of each agent in a centralized way. In this condition, every agent will know the policies of other agents. But the model is executed in a decentralized way. By doing so, every agent can make decision independently.

SABA algorithm uses the same deep *Q*-network algorithm with the proposed algorithm. But the agents of traffic grid don’t communicate with each other. So, every agent doesn’t know traffic condition of other agents. By comparing SABA algorithm with the proposed algorithm, we can test whether considering traffic condition of neighbor intersections is useful for relieving traffic congestion or not. MARDDPG algorithm is also a decentralized ITLC algorithm proposed recently. In this algorithm, agents needn’t to communicate with each other. Every agent can try to know the states of other agents through the model trained in centralized way. Then the optimal policy will be obtained by each agent based on the estimated policy of other agents. The state acquisition method used by MARDDPG algorithm is very distinctive. By comparing MARDDPG algorithm with the proposed algorithm, we can find which kind of decentralized algorithm is more efficient for relieving the traffic congestion.

### Experimental settings

The simulation software used in this experiment is SUMO^36^ (Simulation of Urban MObility). SUMO is an microscopic open-source traffic simulation software. It is discrete in time and continuous in space. Right-side driving rules are supported in this software. Dynamic routing is also supported in this software. And an OpenGL based visual graphical interface is contained in SUMO. The road network used in SUMO can be constructed by either using visual graphical interface or writing XML file directly.

Traffic light used in this experiment contains four phases. Phase 1 is (north, south) & (south, north) green. In this phase, the southbound through and northbound through vehicles can pass the intersection. Phase 2 is (north, east) & (south, west) green. In this phase, northbound left-turn and southbound left-turn vehicles can pass the intersection. Phase 3 is (east, west) & (west, east) green. In this phase, eastbound through and westbound through vehicles can pass the intersection. Phase 4 is (east, south) & (west, north) green. In this phase, eastbound left-turn and westbound left-turn vehicles can pass the intersection. All these phases are mutually exclusive. The length of each road that connects two intersections are 800 meters long. Each road contains three incoming lanes and three outgoing lanes. In every three lanes, leftmost lane is prepared for the vehicles that go straight and turn left. Middle lane is prepared for the vehicles that go straight. Rightmost lane is prepared for the vehicles that turn right.

### Experimental results

**Figure 5:**
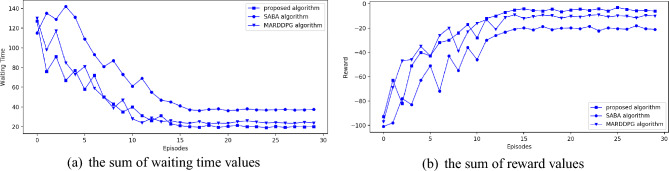
Experimental results based on synthetic dataset.

**Figure 6:**
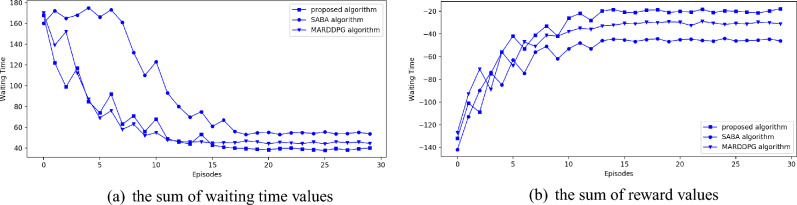
Experimental results based on real world dataset.

Experimental results are presented and discussed in this section. The experimental results obtained based on synthetic dataset are presented in Fig. 5. And the experimental results obtained based on the real-world dataset are presented in Fig. 6. Figures 5a and 6a present the average waiting time of all the vehicles collected at the specific time. In these two figures, the x-axis represents episode number of simulation experiment. There are 30 episodes in this experiment. And each episode contains 200 s. The y-axis represents average waiting time. Moreover, Figs. 5b and 6b present the average reward value of all the agents collected at the specific time. In these two figures, the x-axis also represents episode number of simulation experiment. The y-axis represents average reward value. In all these four figures, line with square on it represents the proposed algorithm. Line with circle on it represents SABA algorithm. Line with triangle on it represents MARDDPG algorithm.

From the experimental results presented in Figs. 5a and 6a, we can find that the average waiting time of all these three algorithms drops while the episode number increasing. Besides, in Figs. 5b and 6b, we can find that average reward value of all the algorithms increases while the episode number increasing. It means that all these three algorithms can relieve traffic congestion. In Figs. 5a and 6a, the curve of SABA algorithm fluctuates at first several episodes. It is mainly because, while executing SABA algorithm, each agent don’t know the traffic condition of other intersections. So these agents can’t take action in advance. Besides, in the first several episodes, the vehicle number increases continuously. So traffic congestion happens frequently in every intersection. Moreover, we can find that the decreasing rate of average waiting time of MARDDPG algorithm is similar to the proposed algorithm, but the curve of proposed algorithm is smoother than MARDDPG algorithm. It means that the information exchange method used in the proposed algorithm is more fit for coping future traffic congestion. Besides, in Figs. 5a and 6a, we can find that average waiting time of these three algorithms will change very little in the last several episodes. And, during this period, waiting time of the proposed algorithm is less than MARDDPG algorithm. Waiting time of MARDDPG algorithm is less than SABA algorithm. It means that the proposed algorithm is better than the other two algorithms in reducing average waiting time of congested vehicles. MARDDPG algorithm is better than SABA algorithm. In Figs. 5b and 6b, the reward values of all these three algorithms are presented. From these two figures, we can find that the reward values of these three algorithms increases while the episode number increasing. And, finally, reward values of these three algorithms fluctuate around a specific value in the last several episodes. Obviously, the curve of the proposed algorithm is smoother than the other two algorithms. It means that the proposed algorithm is more stable than the others. As reward value of the proposed algorithm is higher than MARDDPG algorithm in the last several episodes and reward value of MARDDPG algorithm is higher than SABA algorithm, we can conclude that the proposed algorithm can perform better at increasing reward value. So the proposed algorithm can efficiently reduce both waiting time and queue length of the congested vehicles efficiently.

## Conclusions

In this paper, a new decentralized algorithm is proposed. Traditional decentralized algorithms are mainly concerned with improving the model used by the reinforcement learning algorithm and coordination method. The communication among agents are overlooked. The proposed algorithm tries to design an efficient communication method among agents. Besides, to improve deep *Q*-network model used in the proposed algorithm, a new reward calculation method is proposed in this paper. As traffic congestion can be forecasted based on trajectory, we will try to take laws of vehicle trajectory into consideration while performing traffic signal control in the future research.

## Data Availability

The datasets used and analysed during the current study are available in the https://traffic-signal-control.github.io/#opendatasets.
